# Visual Saliency Models for Text Detection in Real World

**DOI:** 10.1371/journal.pone.0114539

**Published:** 2014-12-10

**Authors:** Renwu Gao, Seiichi Uchida, Asif Shahab, Faisal Shafait, Volkmar Frinken

**Affiliations:** 1 Department of Advanced Information technology, Kyushu University, Fukuoka, Fukuoka, Japan; 2 German Research Center for Artificial Intelligence, Kaiserslautern, Rhineland-Palatinate, Germany; 3 School of Computer Science and Software Engineering, The University of Western Australia, Perth, Australia; University of California Davis, United States of America

## Abstract

This paper evaluates the degree of saliency of texts in natural scenes using visual saliency models. A large scale scene image database with pixel level ground truth is created for this purpose. Using this scene image database and five state-of-the-art models, visual saliency maps that represent the degree of saliency of the objects are calculated. The receiver operating characteristic curve is employed in order to evaluate the saliency of scene texts, which is calculated by visual saliency models. A visualization of the distribution of scene texts and non-texts in the space constructed by three kinds of saliency maps, which are calculated using Itti's visual saliency model with intensity, color and orientation features, is given. This visualization of distribution indicates that text characters are more salient than their non-text neighbors, and can be captured from the background. Therefore, scene texts can be extracted from the scene images. With this in mind, a new visual saliency architecture, named hierarchical visual saliency model, is proposed. Hierarchical visual saliency model is based on Itti's model and consists of two stages. In the first stage, Itti's model is used to calculate the saliency map, and Otsu's global thresholding algorithm is applied to extract the salient region that we are interested in. In the second stage, Itti's model is applied to the salient region to calculate the final saliency map. An experimental evaluation demonstrates that the proposed model outperforms Itti's model in terms of captured scene texts.

## Introduction

In our daily life of the real world, we can almost see texts in any place at any time. While walking at a street, billboards with advertisement texts try their best to be noticed; while driving, traffic signs along the roads provide drivers with information on what to be obeyed; while shopping, labels display the price and other detail of the products. All these indicate that there are many texts in natural scenes.

**Figure 1:**
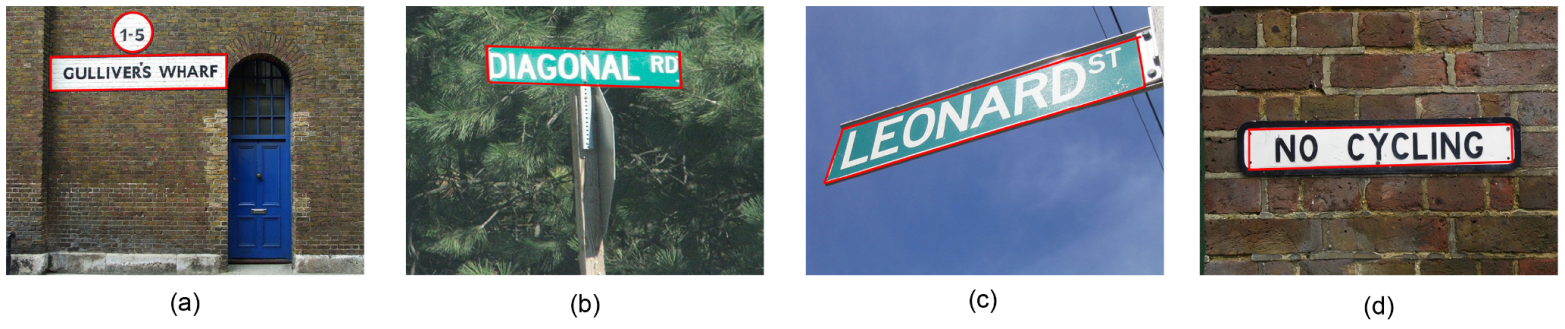
Examples of salient texts (bounded by red lines) in real world. (Copyrights of those figures are listed in Acknowledgments).

The focus of this paper is to analyze the saliency of texts in natural scenes according to different measures of saliency. Scene texts, such as the traffic signal texts and the advertisement texts in the signboards, are considered to convey important information to pedestrians. Therefore they are specifically designed to be prominent to actively attract human's attention. We believe that texts have some kinds of identity properties (e.g. intensity, color or orientation) compared to their non-text neighbors (the so-called pop-up). This is plausible considering that texts in natural scenes, such as those in Fig. 1, are used to communicate important information efficiently to the passengers. In order to make themselves conspicuous, scene texts must be different in some respect of properties. For the quantification of this properties, we will use visual saliency models in this paper.

Visual saliency models describe the observed or predicted behavior of biological primate visual saliency, which describes how the attention of biological primate move while seeing a scene. It has been widely investigated in the field of psychology and computer vision/pattern recognition. Psychologists, neurophysiologists and computer scientists have investigated visual saliency thoroughly during the last decades and profited considerably from each other [1]. Psychologists have studied the behavioral correlates of visual saliency such as change blindness [2], [3], attentional blink [4]. They separated visual saliency into two types: 1) stimulus-driven and 2) task-driven. Neurophysiologists have revealed the biological mechanism of how neurons work with each other to represent the interested objects. Computational scientists have built visual computational models trying to simulate and predict attentional behaviors. Treisman & Gelade [5] proposed the “Feature-Integration Theory” and stated which visual features are important and how they are combined to direct human attention toward pop-out and conjunction search tasks. In 1985, Koch & Ullman [6] proposed a feed-forward model with winner-take-all neural network to select the most salient object and inhibition of return mechanism to shift the focus to the next salient object. Itti *et al.* [7] first implemented Koch & Ullman's model. After that, more than 65 computational models were proposed over the past 25 years [8].

**Figure 2:**
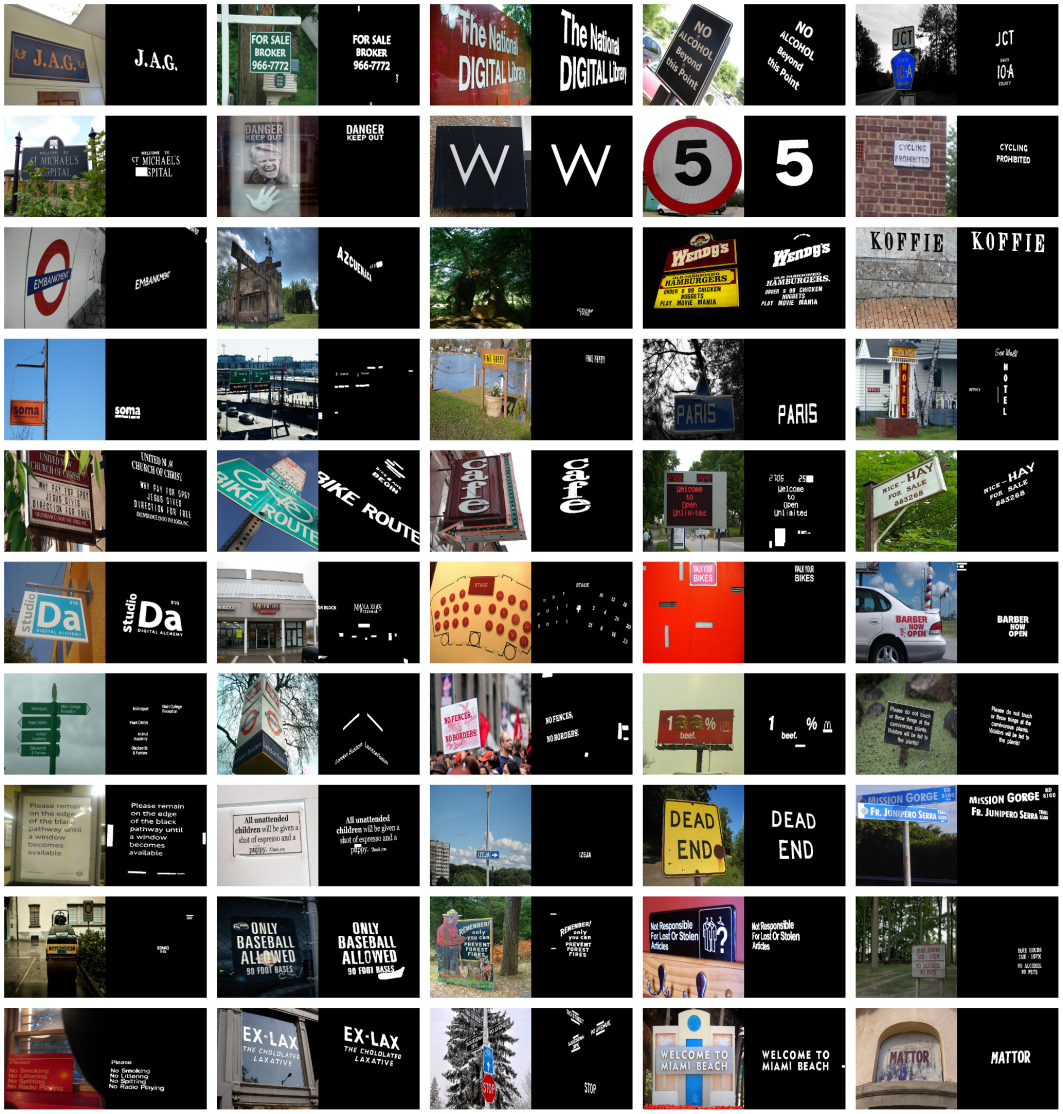
50 examples of images randomly selected from the database. Each example consists of input image (left) and pixel-level ground truth image (right). (Copyrights of those figures are listed in Acknowledgments.)

In this paper, we aim at proving the saliency of scene texts via a large image database which contains 3018 scene images with 96844 text characters totally. Fig. 2 shows some examples of natural scene images and their corresponding pixel level ground truth image randomly selected from the database. With this database and five state-of-the-art visual saliency models, saliency maps are calculated. Saliency values of pixels belonging to texts and non-texts are compared via quantitative evaluation. Hence, if pixels of texts have higher saliency values, scene texts themselves are proven to be salient. The quantitative evaluation will be done using the receiver operating characteristic (ROC) analysis.

If scene texts can be shown to be salient, it will have profound practical implications. For example, it proves that visual saliency models can be used for the task of text detection in natural scenes. Scene text detection is still one of the most difficult tasks to be solved in computer vision because, in addition to the variety of text size and font, the backgrounds of natural scenes can be of arbitrary complexity. Many methodologies have been proposed with the aim of a better detection performance [9]. For example, Coates *et al.* [10] employ a large-scale unsupervised feature learning algorithm to automatically generate features to be used for scene text detection. Mishra *et al.* [11] use Histogram of Gradient (HoG) features and support vector machine (SVM) for the detection of scene texts. Lee *et al*. [12] employ variance and expectation of *x*–*y* derivatives, local energy of Gabor filter, statistical texture, wavelet coefficient and edge interval as features, and use Adaboost algorithm [13] to combine these six features. Epshtein *et al.* [14] use the Stroke Width Transform (SWT) feature, which is able to detect texts regardless of its scale, direction, font and language. Crandall *et al.* [15] develop the discrete cosine transform (DCT) features for scene text detection.

However, only insufficient performance has been achieved. Consequently, if we can show that texts have higher saliency than their background, we will be able to improve those scene text detection methodologies using new feature of scene texts, i.e., saliency, in addition to the conventional features. This expectation is supported by Sun *et al.* [16], Shahab *et al.* [17] and Uchida *et al.* [18], who investigated the effectiveness of visual saliency models for scene text detection based on rather small scale database (for example, only 300 images are used in [17] with limited investigations).

The main contributions of this paper can be summarized as follows: 1) The first trial of employing visual saliency for proving the saliency of scene texts using large scale scene image database with pixel level ground truth is provided, and 2) a new model of hierarchical saliency which captures more scene texts than conventional model is proposed.

## Visual Saliency Models

In the past 25 years, over 65 kinds of visual saliency models have been proposed [8], most of which might be able to evaluate the saliency of scene texts. Visual saliency models can be roughly classified into three catalogues: a) Bottom-up model, b) top-down model, and c) hybrid model. The bottom-up model of visual saliency [7], [19] considers three low level channels (intensity, color and orientation) as the feature to identify the salient locations. It consists of three stages, including 1) *feature maps calculation*, 2) *conspicuous maps calculation*, and 3) *saliency map calculation*. Bottom-up model of visual saliency is task-independent as it does not use any prior information (such as the size and/or shape of the object) to identify the salient objects. In contrast to bottom-up model, top-down model of visual saliency is task-dependent and use prior contextual knowledge to guide to the objects. This kind of model is based on the fact that the context of the scene governs how a person's attention changes while searching for an object [20]. For example, while scanning for pedestrians, we mainly focus our attention on the bottom of the scene and pay less attention on the top part. However top-down model requires at least a basic image understanding technology, which is still a difficult problem to be solved. The hybrid model of visual saliency combines a bottom-up and a top-down model by using a Bayesian framework [8]. The top-down model is used to indicate the probability of finding the target at the given place, while the bottom-up model verifies the target.

**Figure 3:**
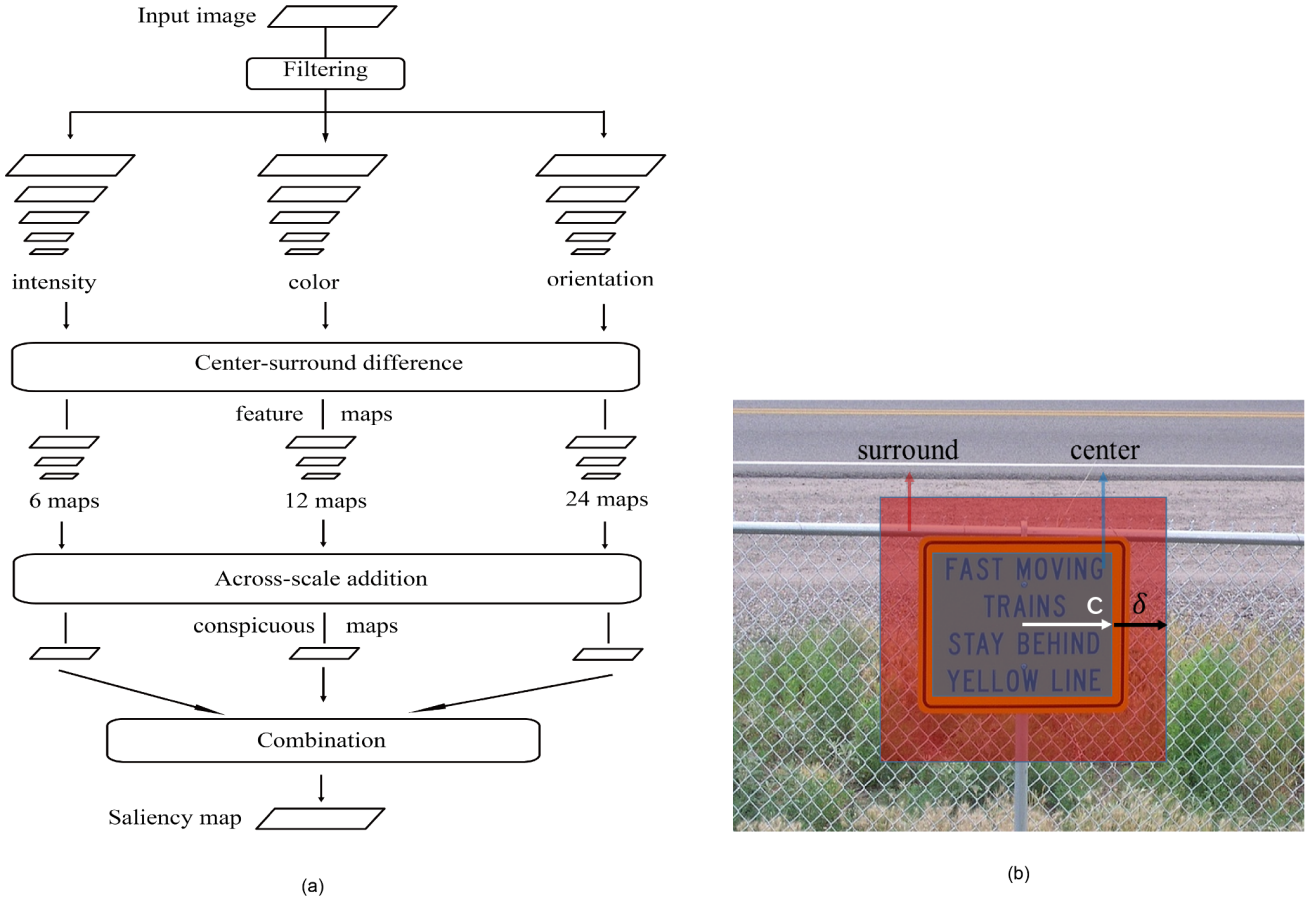
Itti's visual saliency model. (a) Itti's visual saliency architecture [7]. (b) Center-surround diagram. (Copyrights of those figures are listed in Acknowledgments.).

In 1998, inspired by the behavior and the neuronal architecture of the early primate visual system, Itti *et al.* [7] implemented the first complete visual saliency model of Koch & Ullman [6]. This model uses three low level channels (color, intensity and orientation) as features and calculates saliency map, which is defined as the degree of difference between an object and its neighbors, for each channel. The degree of difference is measured by the center-surround operation, which is implemented as the subtraction of images that derive from the same image and are at different scales. In this implementation, Itti *et al.* down-sampled image from 1 (1∶1) to 8 (1∶256) scales, and defined images at 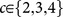 scales as center and images at *s* = *c*+*δ* scales, with 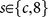 for each c and 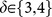, as surround, which is experimentally proved that can maximize the difference. Fig. 3(b) illustrates the meanings of parameter *c* and *δ*. The final saliency map is obtained by combining the three saliency maps.

Like Itti's visual saliency model, Harel *et al.*'s [19] graph-based visual saliency model (GBVS) utilizes the same features; unlike Itti's visual saliency model, it makes use of the self-information (entropy) of the object instead of using the simple center-surround operation while processing the feature maps and combining feature maps into activation maps (conspicuous maps) and final saliency map. In order to calculate the self-information, a Markov chain is applied to construct the full connected directed graph which joins all pixels (nodes). Weight of each edge is defined as the dissimilarity and the distance of the two pixels. The more dissimilar as well as the further apart two pixels are, the smaller is the value of the weight. Equilibrium distribution is employed to ensure that, for a given pixel (node), the total weights of the outbound edges is 1. The saliency of the pixel is calculated as the self-information via Shannon formula.

**Figure 4:**
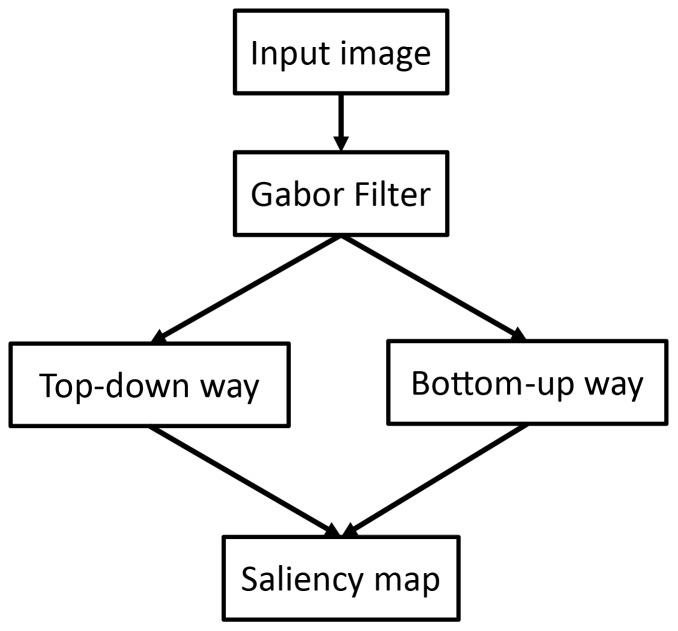
Simplified architecture of Torralba's contextual guidance model. For the complete architecture and more details, please refer to [20].

Combining visual saliency with statistical methods, Torralba *et al.* [20] proposed a hybrid visual saliency model. This model uses Gabor filters to calculate the orientation features (local features) based on which GIST and principal component analysis (PCA) technologies are used to calculate global features for distinguishing different natural scenes. It consists of two components: 1) bottom up component, which is defined as the inverse of the probability of finding the given local feature under the given natural scene and is used to evaluate the saliency, and 2) top down component, which is trained to classify where the object may be by using global features and Gaussian mixture model (GMM), and is used to filter objects that are less like the target. The final saliency map results from the product of the two components. Fig. 4 shows the details of the architecture of Torralba's contextual guidance model.

Zhang *et al.* [21] also proposed a hybrid visual saliency model based on Bayesian framework. This model aimed at finding potential targets that might be important for survival, such as food and predators. Unlike the above mentioned models, in which model the saliency of an object is calculated based on the current viewing image, this kind of visual saliency model calculates the saliency of an object based on the images that have been viewed before (past experience). It defines the bottom up saliency of a given object as the probability of it having been seen in the past, that is, the less rare been seen, the more salient it is. This definition is reasonable considering the problem of survival, especially in a dangerous situation. The prior top down knowledge is defined as how the object is similar with the target. All probability distributions are trained off-line on a prepared natural scene image database. The final saliency map is constructed via combining the bottom up saliency component and the top down component. We use Nich *et al.*'s [22] C++ implementation of fast saliency, which is optimized for robot vision by the use of difference of box (DoB) filters and estimating a Laplacian distribution of unit variance. In the later parts of this paper, we use the term fast saliency to represent Zhang's visual saliency model.

Achanta *et al.* [23] proposed a novel saliency model in the frequency domain for saliency region detection. This model is mainly to find the largest salient objects in a full resolution (the same resolution as the original input image), in which all the salient regions are uniformly highlighted. All the requirements are satisfied by feeding two thresholds to the difference-of-Gaussian (DoG) filter: 1) *w_lc_* for cutting off the low frequency to emphasize the largest saliency objects, and 1) *w_hc_* for disregarding high frequencies that are raised from texture, noise and blocking artifacts. The saliency map is calculated as the absolute difference between the arithmetic mean pixel value of the original image and the DoG filtered original input image. The reason to include Achanta's frequency-tuned model in this study are two-fold: first, it obtained the best results in [23]; second, it is based on a different theoretical foundation than the other four models.

## Visual Saliency of Scene Texts

As noted before, texts in advertisements are often written on top of a simple background to let the product appear more impressive as well as to improve readability. Similarly, to emphasize sentences in an article, we make them colorful or italic to be noticed easily. As a general observation, texts in natural scenes are intended to advertise something or navigate people (e.g. texts in traffic signs). In short, scene texts are designed to draw attention. Therefore scene texts should give good responses to visual saliency models. In this section, the mentioned five state-of-the-art visual saliency models are employed to calculate the responses to texts in natural scenes.

### Database

The database we used throughout this paper was prepared by our laboratory. It contains 3018 natural scene images, collected from the website “flickr” (https://www.flickr.com). Each image has a maximal size of 640×640 and a minimal size of 223×240. The entire database contains 96844 characters totally. For each image of the database, the ground truth image was created by manually labeling the pixels belonging to texts, as shown in Fig. 2.

### Evaluation Protocol

Whether scene texts have higher visual saliency value than non-text regions is evaluated in the following scene text detection task. In the experiment, for each saliency map *S*, pixels are classified as belonging to texts if, and only if, their visual saliency values were higher than the given threshold 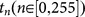. Given the corresponding ground truth image *I_GT_* with a number of text pixels *G_T_* and a number of non-text (background) pixels *G_B_*, the text detection accuracy at threshold *t_n_* is evaluated as follows:

- The number of pixels in both saliency map *I*′ (salient pixels) and ground truth *I_GT_* (text pixels), 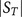;
- The number of pixels that are salient in the saliency map *I*′, but belong to the non-text regions in the ground truth image *I_GT_*, 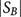.

For each threshold, the following performance metrics are calculated:
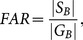
(1)and
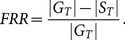
(2)


In the experiments, performances are evaluated using receiver operating characteristic (ROC) curves. The false acceptance rate (FAR) is plotted against the false rejection rate (FRR) for all values of the threshold *t_n_*. The point on the curve closest to the origin represents the best performing algorithm as it has the lowest equal error rate. Note that we use C++ source code from Neuromorphic Vision C++ Toolkit (iNVT), which is developed at iLab, USC (http://ilab.usc.edu/toolkit), for Itti's visual saliency model to calculate saliency map, and the Matlab implementation for Harel's graph-based visual saliency model (http://www.klab.caltech.edu/~harel/share/gbvs.php) and Achanta's frequency-tuned salient region detection model (ivrgwww.epfl.ch/supplementary_material/RK_CVPR09).

## Results and Discussion

**Figure 5:**
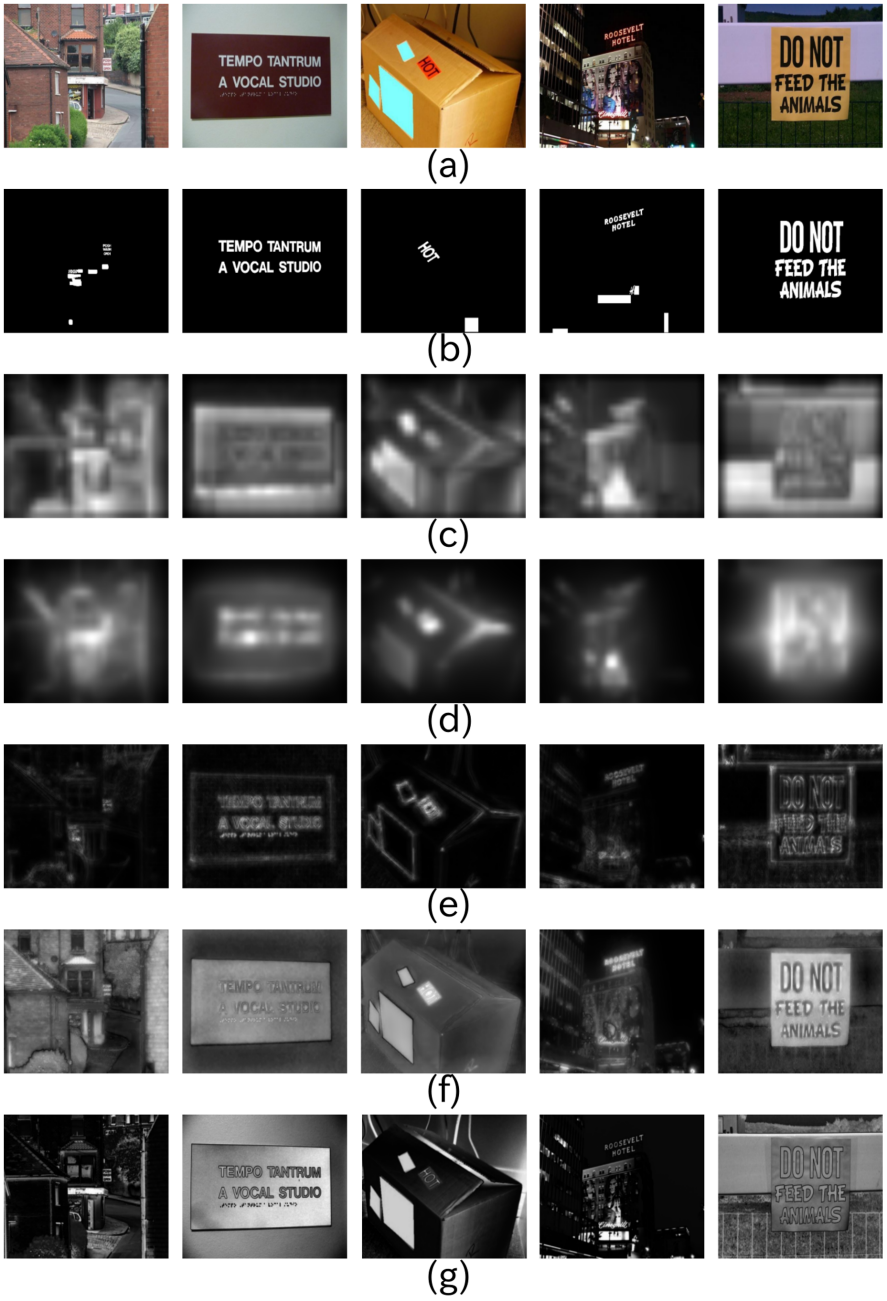
Examples of five state-of-the-art saliency maps. (a) Input images. (b) Ground truth images. Rectangles mean “don't care” texts. (c) Visual saliency maps from Itti's visual saliency model. (d) Visual saliency maps from Harel's graph-based visual saliency model. (e) Visual saliency maps from Torralba's visual saliency model. (f) Visual saliency maps from Fast saliency model. (g) Visual saliency maps from frequency-tuned model. For more examples, please refer to Fig. 15. (Copyrights of those figures are listed in Acknowledgments.)

**Figure 6:**
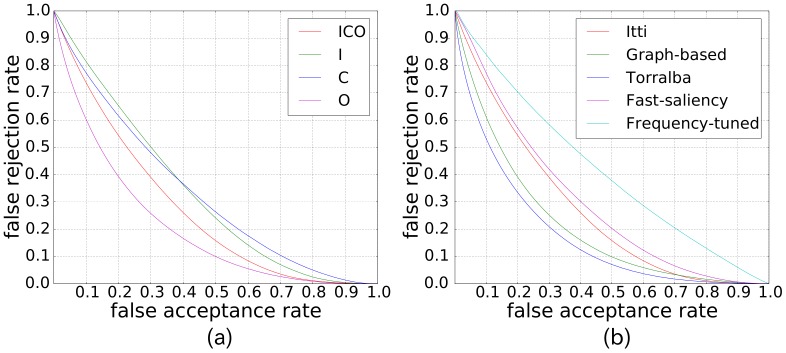
How salient are scene texts. (a) Performance evaluation of Itti's visual saliency model with different parameters. The letters I/C/O represent Intensity/Color/Orientation, respectively. (b) Performance evaluation of five state-of-the-art visual saliency models.

Three experiments are given in this section. The first experiment (Fig. 5) was done to calculate the saliency maps using the five state-of-the-art visual saliency models with the aim of evaluating how scene texts are salient qualitatively. The second experiment (Fig. 6(a)) is the ROC-based performance evaluation of Itti's visual saliency model with different features. This experiment was done in order to investigate how salient scene texts are for each low level feature. Finally, the last experiment (Fig. 6(b)) is the ROC-based performance evaluation, using all the five state-of-the-art visual saliency models.

Are scene texts salient? The first experiment gives a positive answer to this question. Fig. 5 shows, although the five state-of-the-art models gave different responses to the same input scene images, they responded well to scene texts. In the case of Itti's visual saliency maps (c) and Harel's graph-based visual saliency model (d), scene texts seem to be more salient compared to the non-texts, while the non-texts were not well inhibited. This might be, due to the fact that both Itti's visual saliency model and Harel's graph-based model are task-independent and stimuli-driven. They focus mainly the difference of an object and its neighbor regardless if it is scene text. In the case of Torralba's saliency maps (e), all texts are salient, and to some extent, compared with saliency maps from Itti's and Harel's graph-based visual saliency models, the saliency of non-texts were well inhibited. In the saliency maps using the fast saliency model (f), scene texts appear less salient. Achanta's model (g) prefers the high frequency texts, which is unfortunately not the common case in natural scenes.

**Figure 7:**
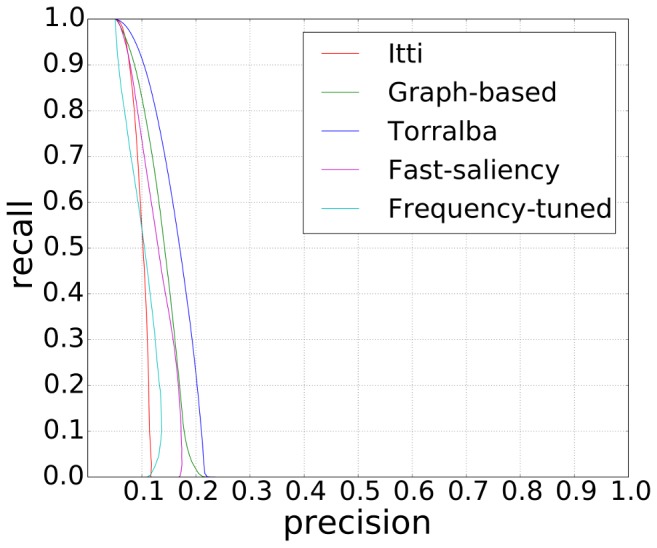
Text saliency evaluation using precision and recall rate measure. This does not mean texts are not salient. The reason for the low precision is that, our evaluation is pixel-level based and pixels belonging to texts are far less than those belonging to non-texts in natural scene images. This results in, while evaluating the degree of visual saliency of texts for a given degree (e.g. 200 with respect to pixel value from 0 to 255), pixels belonging to texts taking few percentage.

**Figure 8:**
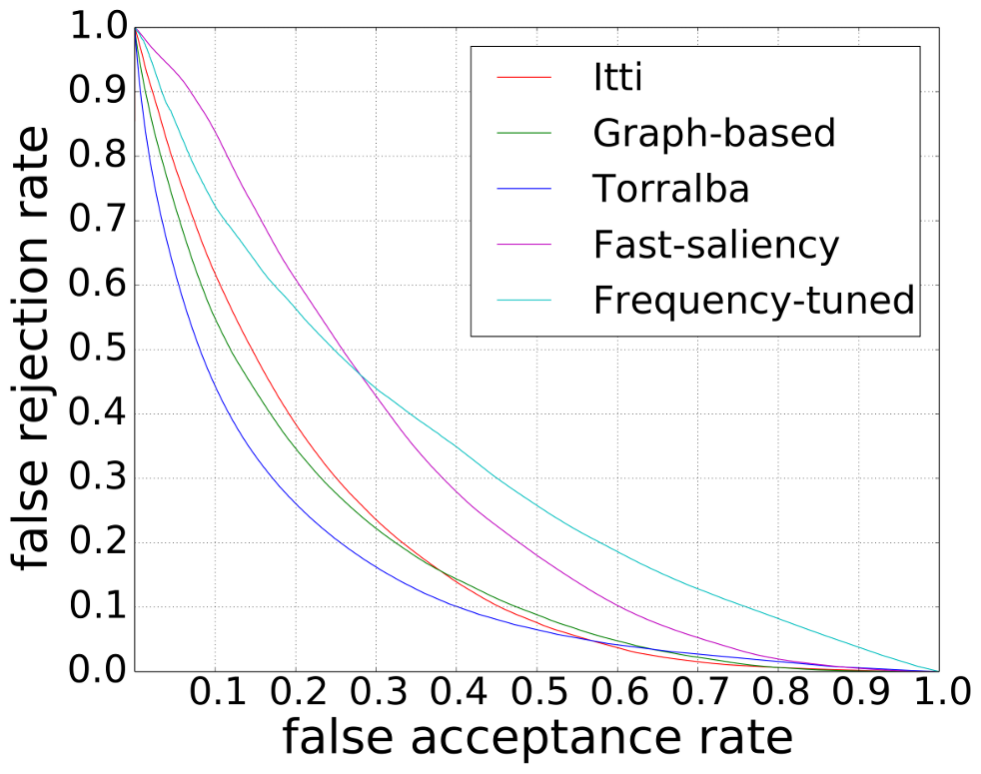
Text saliency evaluation on ICDAR 2011 image database. The results we obtained are similar to those using our database. This indicates that texts are salient regardless of the database we are using.

The second and third experiments show how salient scene texts are. Fig. 6(a) shows the saliency of scene texts using Itti's visual saliency model with different low level features. It indicates that the orientation channel gives the best response to scene texts. This is mainly because scene texts are generally written in a simple board, who have smooth surfaces with few orientation features, whereas the texts have strong orientation features, such as edges, etc. Color and intensity-based features perform almost the same. From Fig. 6(b), we know that Torralba's visual saliency model gave the best response to scene texts, while the frequency-tuned model performed the worst. This is because Torralba's model can not only response well to scene texts, but also prevents the non-texts from being salient. Frequency-tuned model, on the contrast, aimed to segment the largest high frequency objects from scene images rather than to find the salient pixels. Fast saliency model also gave low saliency value to scene texts, indicating that texts are not rare in natural scenes. Though Harel's graph-based visual saliency model obtained not as good performance as Torralba's visual saliency model, it had a better result than Itti's visual saliency model. The explanation to this fact is that Harel's graph-based visual saliency model can pay more attention on texts rather than the background. Note that besides using the ROC curves and our database, we also evaluated the degree of text saliency using the precision and recall rate measurement (see Fig. 7), and the standard ICDAR 2011 database (see Fig 8), which gave the similar result. Though Torralba's visual saliency model achieved the best result, we only are interested in the task-independent model [24]. For the following discussion and calculation, we use Itti's visual saliency model rather than all the five state-of-the-art models.

**Figure 9:**
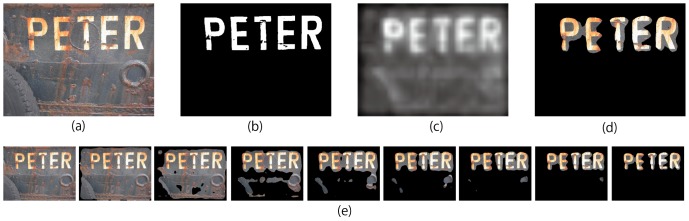
How scene texts are detected using Itti's visual saliency model with intensity, color and orientation. (a) The input scene image. (b) The ground truth image. (c) Saliency map. (d) Detected scene texts with a certain threshold. (e) Changes of the detection results under different threshold values. From left to right, threshold is 0, 20, 40, 60, 70, 80, 90, 100 and 120. (Copyrights of those figures are listed in Acknowledgments.).

As a simple example of applying visual saliency models to scene text detection, Fig. 9 demonstrates how scene texts can be detected. In the set of result images (Fig. 9(e)), each image was obtained as follows: the saliency map (Fig. 9(c)) was binarized by applying a set of threshold ranging from 1 to 255 (for each binarized image, 0 represents non-texts, and 1 represents texts); result image was obtained by multiplying the binarized image with the original input scene image.

**Figure 10:**
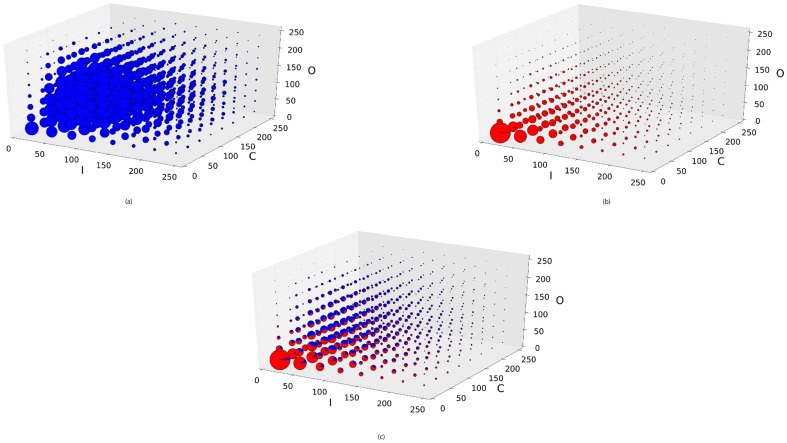
Distribution of scene texts and non-texts in the space constructed by intensity, color, and orientation. (a) Distribution of intensity, color, and orientation saliency of pixels that belong to texts. (b) Distribution of intensity, color, and orientation saliency of pixels that belong to non-texts. (c) Distribution of pixels from both texts and non-texts. For those plots, 200 pixels (100 pixels from texts and 100 pixels from non-texts) are randomly selected from each saliency map.

In order to visualize why texts can be detected from scene images, we plotted the distribution of scene texts and non-texts in the space that constructed by three kinds of saliency maps which were calculated using Itti's visual saliency model with intensity, color, orientation feature respectively. Fig. 10 shows the distribution of scene texts and non-texts. For each subfigure, each axis is split into 8 bins (8 = 256/32) and the whole space is split into 512 cells (8×8×8) evenly. For each cell, the size of chart (the circle) represents how many pixels distribute in the cell. The blue parts of the charts represent pixels of scene texts, and the red ones represent pixels of non-texts. Fig. 10 shows that pixels belonging to non-texts almost distributed at the low value of intensity, color and orientation, while pixels belonging to texts mainly distributed at the cells with higher values. All these results indicate that 1) Scene texts are visual saliency, and 2) visual saliency models can be used for scene text detection. For the latter point, it is favourable that the calculation of visual saliency is generally very fast (less than one second per entire image for Itti's method, only 150 ms on our computer with Intel Xeon CPU). (We evaluated the time complexity for the above mentioned models, which indicates all of the models can be used for real time application. For example, Itti's visual saliency model costs only 0.165 second per image on average. Since our model runs twice of Itti's method, it costs 0.32 second per image. We can even boost the execution of our purposed hierarchical visual saliency model by parallelizing the two stages in the case that the input is a sequence of frames rather than a single image.)

### Hierarchical Visual Saliency Model

In this section, first a new assumption is formulated based on the observation of the qualitative evaluations. Then, a new visual saliency model, named “Hierarchical Saliency Model” is proposed according to the assumption. Finally, a novel visual saliency model is presented, based on the idea of cascade structure calculation.

### A New Assumption

From the observation of Fig. 5 we can observe that in some situation (e.g., in the second saliency map of Fig. 5(c)), pixels belonging to the texts themselves are less salient than expected; instead, “text carriers” onto which scene texts are written (such as the signboards or billboards) are salient enough by themselves to attract one's attention. This fact reveals that in some situation texts are not salient when seen in the context of the entire image. However, when we consider only the signboards region (or other objects onto which the texts are written) texts are salient. This means that “text carriers” seems to be designed to be salient globally (compared to other parts of the image), whereas the texts seem to be designed to be salient locally (compared to their near non-text neighbors).

Based on the above observation, a new assumption is made: texts are prominent comparing to their near non-text neighbors, although they may not be in a global view of the image. It means that texts might be often locally salient inside their possibly globally salient regions. Taking the car license number plate for example, texts on the car license number plate might be less prominent than the license number plate if we put our attention on the whole car, however, they become highlighted in the case we put eyes only on the number plate.

### Hierarchical Saliency Model

**Figure 11:**
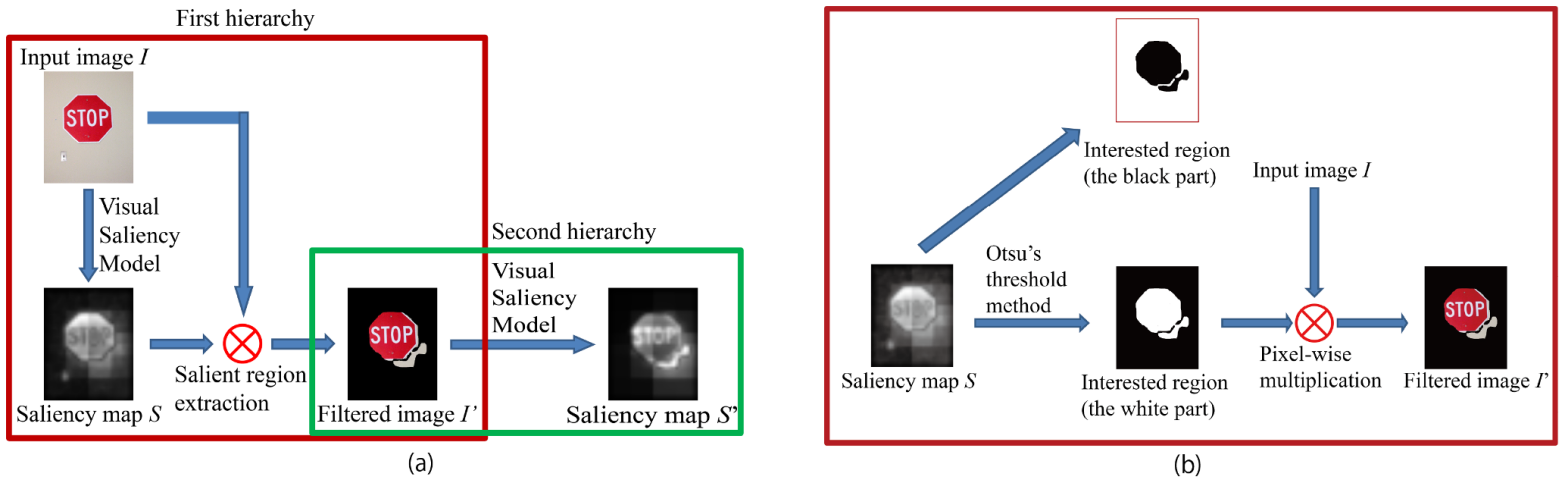
Architecture of hierarchical visual saliency model. (a) The procedure of hierarchical visual saliency model. (b) Details of salient region extraction. (Copyrights of those figures are listed in Acknowledgments.).

A new approach for text detection in natural scenes is proposed based on this new assumption. We call it *“Hierarchical Saliency Model”* because its final saliency map is calculated hierarchically (or say iteratively, see Fig. 11):

- First hierarchy (extraction of globally salient region):
  1. Calculate the saliency map *S* from the given image *I*;
  2. Evaluate the globally salient region from *S*. All pixels of *S* are automatically classified into two categories to obtain mask image *M*: the globally salient region (assigned to 1) and the rest (assigned to 0);
  3. Multiply the mask image *M* with the input image *I* to calculate filtered image *I*′;
- Second hierarchy (evaluation of local saliency inside the globally salient region):

   Use *I*′ to obtain a new saliency map *S*′, which is the final map we want.

Note that though we use the same saliency model to calculate the saliency map in both first and second hierarchies, the saliency values might be different even for the same pixels. This is simply because the areas subjected to the model are different.

In this paper, the Otsu's global thresholding method [25] and the simple merging-based Ward's hierarchical clustering method [26] were employed for salient region extraction, while those methods provided similar results. We employ Itti's visual saliency model, instead of all the models, for the following calculation and discussion, not only because we are only interested in the task-independent visual saliency model, but also because Itti's visual saliency model is the first complete implementation of bottom-up model.

### Experiment and Discussion

**Figure 12:**
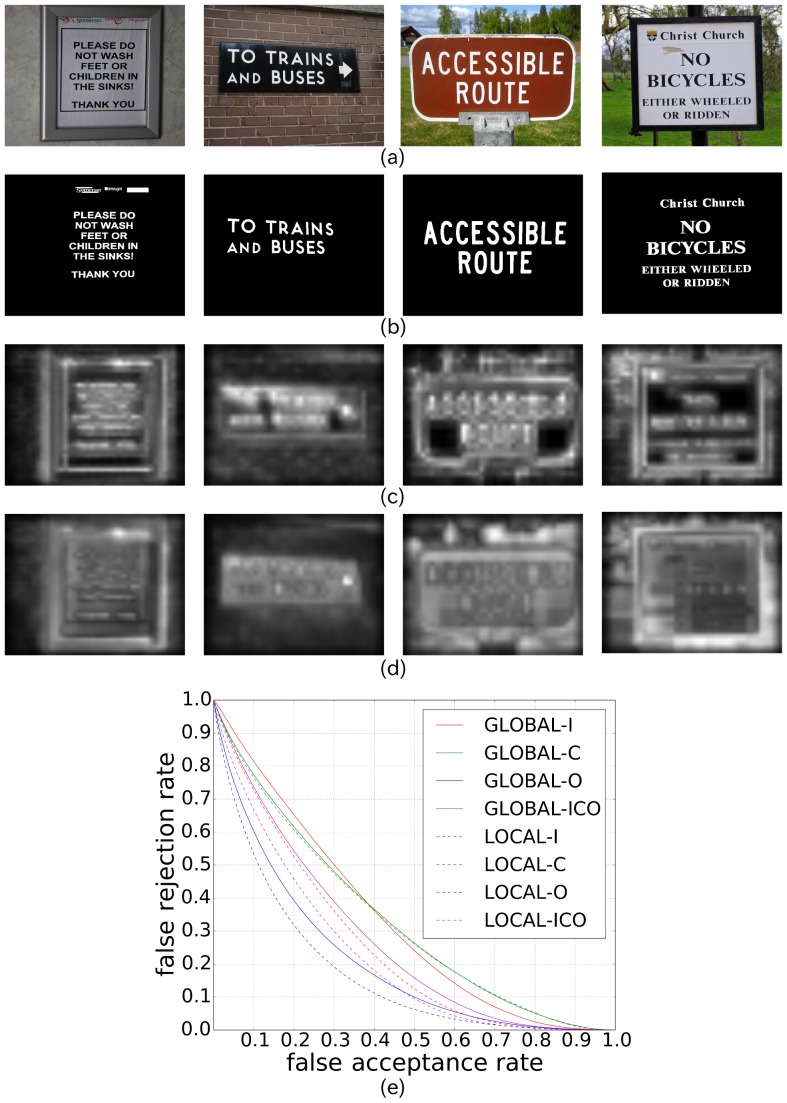
Local saliency vs. conventional Itti's saliency of scene texts. (a) The input images. (b) The corresponding ground truth. (c) Local saliency maps (set *δ* to 1). (d) Conventional Itti's saliency maps. (e) ROC curve comparison of local saliency and Itti's saliency. (Copyrights of those figures are listed in Acknowledgments.).

In order to validate the assumption that “texts are more locally salient than globally salient”, an experiment was done by setting the parameter *δ* of Itti's visual saliency model to 1. In the stage of center-surround difference in the architecture of Itti's model, for a given center *c*, *δ* controls which surround to compare to. For small values of *δ*, the saliency of the center is considered only within a local context [7]. Comparing Fig. 12(c) with Fig. 12(d) we can know that scene texts were more salient by setting *δ* to 1. Fig. 12(e) shows that for each low level channel of Itti's visual saliency model, scene texts were more locally salient than globally salient. This means that the assumption we made is reliable, and the hierarchical visual saliency model is also a worth of trial.

**Figure 13:**
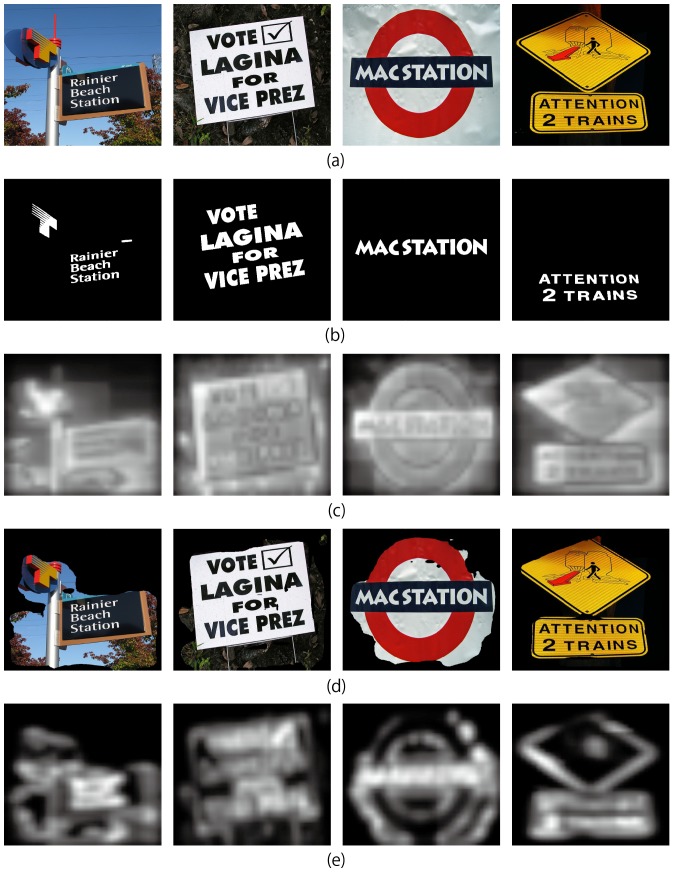
Results of hierarchical saliency model. (a) Input images. (b) Ground truth images. (c) Itti *et al*.'s visual saliency maps, calculated from (a) with all the low level features. (d) Salient region images extracted from (c) using Otsu's global thresholding algorithm. (e) Itti *et al*.'s visual saliency maps, calculated from (d). (Copyrights of those figures are listed in Acknowledgments.).

**Figure 14:**
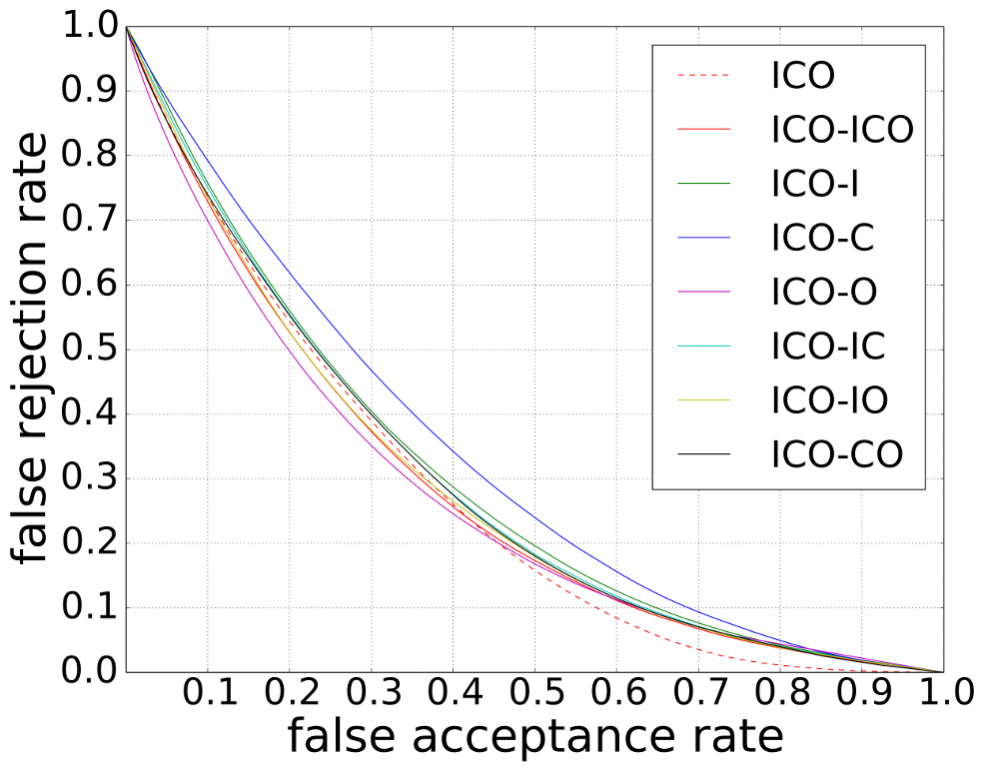
Comparison between hierarchical visual saliency model and the conventional Itti's visual saliency model. We use Otsu's global thresholding algorithm to extract the salient region (according to our experiment, the Ward's hierarchical clustering method gave the similar results). The letter I is short for Intensity, C represents for Color, and O means orientation.

Experiments using the Otsu's global thresholding algorithm for the salient region extraction were held in order to compare the conventional Itti's visual saliency model with the proposed model. Fig. 13(c) shows the saliency maps of the conventional Itti's visual saliency model using all the low level channels. It shows that scene texts were not salient enough. Yet, the “text carriers” are salient. Fig. 13(d) shows the salient regions extracted using the Otsu's global thresholding algorithm. Though the extracted regions included the extra parts that do not carry texts, all the texts were extracted. Fig. 13(e) shows the hierarchical visual saliency maps. It can be seen that scene texts are more salient than their neighbors using the hierarchical visual saliency model. Fig. 14 depicts the comparison between the conventional Itti's visual saliency model and the hierarchical visual saliency model. From the figure, we can see that the proposed hierarchical visual saliency model can capture more scene texts than the conventional Itti's visual saliency model. The proposed model achieved this by inhibiting the less salient regions which are considered less possible to be texts, and focusing on the salient regions which are more likely to include texts.

## Conclusion

Two main contributions have been achieved in this paper. Firstly, we demonstrate through a set of experiments that the saliency of scene texts using large scale scene image database with pixel level ground truth, and secondly, a new model of hierarchical saliency model which captures more scene texts than conventional model was proposed.

We conducted the first large scale experiment with pixel level ground truth for showing that scene texts are salient. Five state-of-the-art visual saliency models were employed to investigate the saliency of scene texts, and two quantitative performance evaluation were given. Evaluation on the contribution of the three low level channels of Itti's visual saliency model to the saliency of scene texts revealed that the orientation channel contributes the most to the saliency of scene texts, while the color and the intensity channels contribute the same. According to the performance evaluation of the five stat-of-the-art visual saliency models, we conclude that Torralba's contextual guidance model gave the best response to scene texts. In contrast, Achanta's frequency-tuned visual saliency model performed the worst. A visualization of the distribution of scene texts and non-texts is also shown. From the visualization of distribution we can conclude that scene texts can be captured from the scene images.

We made a new assumption, and, according to this assumption, proposed a new visual saliency model named hierarchical saliency model. We found that sometimes, the text “carriers”, in which texts are written, are more salient than texts themselves; however when we focus our attention on these “carriers”, texts become salient. This indicates that texts in natural scenes are more locally (comparing to their near neighbors) salient than globally (comparing to the entire scene) salient. An experiment was done by setting *δ* to 1 to validate this assumption, and proved that the assumption is reliable. Also, a quantitatively performance evaluation between the conventional Itti's visual saliency and the proposed model was given. This experiment shows that the proposed hierarchical visual saliency model captures more scene texts than the conventional Itti's model.

## Supporting Information

S1 Figure **More examples of saliency maps of the five state-of-the-art models.** (Copyrights of those figures are listed in Acknowledgments.).
(TIF)
