# Supplementary figures and images for: Visual Saliency Models for Text Detection in Real World
Source: PLoS One. 2014 Dec 10;9(12):e114539. doi: 10.1371/journal.pone.0114539 (PMC4262416; doi:10.1371/journal.pone.0114539)

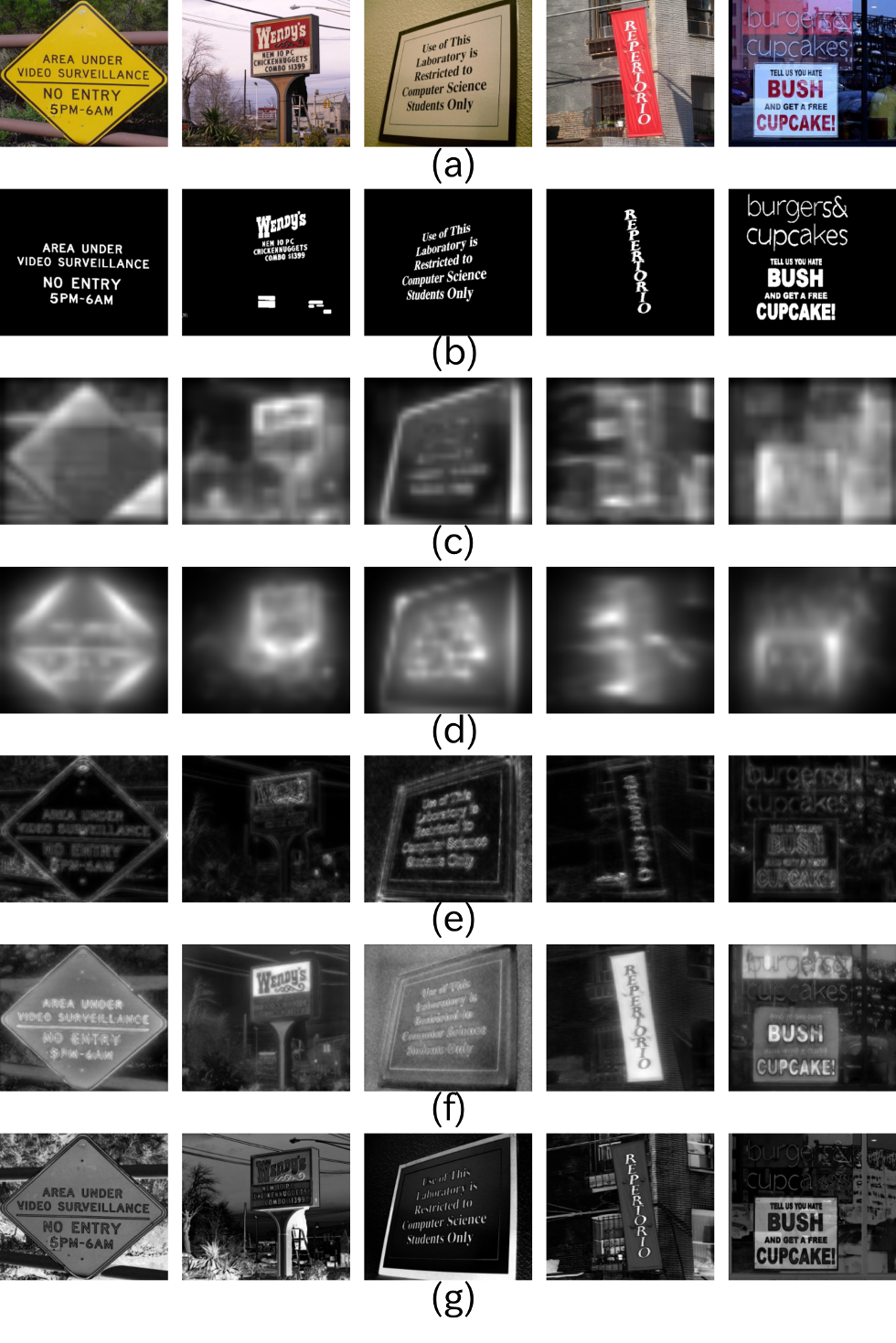

Supplement: S1 Figure — More examples of saliency maps of the five state-of-the-art models. (Copyrights of those figures are listed in Acknowledgments.). (TIF) [file pone.0114539.s001.tif]
